# Proteomic analysis reveals biomarkers associated with performance-based joint function and patient-reported outcomes in knee osteoarthritis

**DOI:** 10.1016/j.ocarto.2024.100543

**Published:** 2024-11-16

**Authors:** Josefine E. Naili, Aisha S. Ahmed, Margareta Hedström, Morten Bilde Simonsen, Eva W. Broström, Helena Erlandsson Harris, Ákos Végvári, Cecilia Aulin

**Affiliations:** a Dept. of Women’s and Children’s Health, Karolinska Institutet, Karolinska Vägen 37 A, QA 02:07, 171 76, Stockholm, Sweden; b Motion Analysis Lab, Karolinska University Hospital, Stockholm, Sweden; c Dept. of Molecular Medicine and Surgery, Center for Molecular Medicine, Karolinska Institutet, Stockholm, Sweden; d Dept. of Clinical Science, Intervention and Biotechnology, CLINTEC Karolinska Institutet, Stockholm, Sweden; e Trauma and Reparative Medicine Theme (TRM), Karolinska University Hospital, Stockholm, Sweden; f Dept. of Materials and Production, Aalborg University, Fibigerstræde 16, 9220, Aalborg, Denmark; g Center for Mathematical Modeling of Knee Osteoarthritis, Aalborg University, Denmark; h Dept. of Medicine Solna, Division of Rheumatology, Centre for Molecular Medicine, Karolinska Institutet, Karolinska University Hospital, Stockholm, Sweden; i Broegelmann Research Laboratory, Department of Clinical Science, University of Bergen, The Laboratory Building, 5th Floor, Haukeland University Hospital, Jonas Lies Vei 87, N-5021, Bergen, Norway; j Dept. of Medical Biochemistry and Biophysics, Karolinska Institutet, Biomedicum, Solnavägen 9, 171 65, Stockholm, Sweden

**Keywords:** Mass spectrometry, Plasma, Biomechanics, Kinematics, Inertial measurement units, Patient-reported outcome measure: performance-based function

## Abstract

**Objective:**

This study aimed to identify proteins associated with clinical manifestations of knee osteoarthritis (KOA), including performance-based joint function and patient-reported outcome measures (PROM).

**Methods:**

This cross-sectional exploratory study included thirteen individuals with KOA and eleven age-matched controls. All participants performed the 30s Single Leg Mini Squat test and 30s Sit-to-Stand test with simultaneous recording of joint kinematics. Individuals with KOA completed the Knee Injury and Osteoarthritis Outcome Score and Forgotten Joint Score-12. Proteins were determined by quantitative mass spectrometry (MS) in plasma. Principal component analysis (PCA), hierarchical cluster analysis (HCA), and Reactome enrichment analysis of the proteome were conducted to identify activated pathways and groups.

**Results:**

Performance-based function was worse in individuals with KOA compared to controls, and they reported higher levels of pain. MS analysis identified 82 differentially expressed proteins (DEPs) in KOA (28 upregulated, 54 downregulated of 321 detected proteins). PCA displayed distinct features between KOA and controls, similar to HCA, which distinguished two major clusters. Enrichment analysis displayed platelet activation and degranulation, neutrophil, and extracellular matrix (ECM)-related pathways. From the proteome, 23 DEPs were associated with different aspects of joint function, and 25 DEPs with PROM.

**Conclusions:**

Individuals with KOA differed from controls across all three assessment modalities; they presented worse joint function, higher levels of pain, and an altered plasma protein profile. Multiple associations were observed between up- and downregulated DEPs and clinical manifestations. The described study protocol shows promise for performing multivariate analyses for future subgrouping of individuals with KOA.

## 1 Introduction

Knee osteoarthritis (KOA) is a common form of arthritis, causing joint pain, reduced range of motion (ROM), and muscle weakness. This leads to functional impairments, limited activities of daily living [1,2], and reduced physical activity [3]. KOA is a multifactorial disease where the precise underlying causes are not fully understood [4].

Biomarkers in OA have been extensively studied, with structural proteins from cartilage, as well as inflammation- and coagulation-related proteins, being proposed for diagnostic or prognostic use [[5], [6], [7], [8]]. However, none of these candidate biomarkers have been introduced into clinical practice. Recent advances in proteomic and multi-omic approaches show promise in elucidating the molecular pathways driving OA progression [8]. For biomarkers to be clinically useful, they must correlate with patient characteristics and relevant clinical measures. Previous studies have linked protein levels to demographic factors such as age, sex, BMI, and radiological assessments [9], while others have considered patient-reported outcomes measures (PROM) of pain [10]. Objective methods to evaluate joint function provide additional information, not necessarily influenced by pain, by capturing subtle changes in joint function [11,12]. When combined with molecular data, assessments of joint function may help establish criteria for defining or predicting disease progression [13]. Performance-based measures of joint function and PROM are only moderately correlated, as they measure different constructs, making them complementary tools in patient evaluation [14]. Both measures are therefore important for evaluating patients with KOA [15]. Integrating molecular markers with objectively assessed joint function and PROM could pave the way for new criteria to predict disease progression.

The BIOFUNC study aims to investigate the connections between biomarkers and performance-based joint function, as well as patient-reported function and pain in OA. This study presents data from BIOFUNC, correlating mass spectrometry (MS) proteomics with two critical clinical issues in KOA: reduced joint function and patient-reported pain and symptoms. This exploratory, hypothesis-generating investigation primarily aimed at identifying differentially expressed proteins (DEPs) in patients with KOA compared to non-pathological individuals and examining their associations with performance-based joint function and PROM. The secondary aim was to explore specific molecules and pathways activated or dysregulated in KOA. The BIOFUNC study setup represents an initial step toward mapping KOA and facilitating future disease subtyping.

## 2 Materials and methods

### 2.1 Study design and study participants

The study is in accordance with the Helsinki Declaration, and ethical approval was obtained from the Swedish Ethical Review Authority (Dnr: 2019-02009). Strengthening the Reporting of Observational Studies in Epidemiology initiative was followed in the reporting of this study [16]. All study participants received oral and written information about the study procedures and provided written informed consent prior to study participation. The data included in the study was collected between March 2021 to June 2022.

Individuals with symptomatic KOA were recruited from local orthopedic departments and one out-patient clinic in Stockholm, Sweden. Inclusion criteria included the American College of Rheumatology diagnosed KOA [17], the ability to walk 10 ​m repeatedly without a walking aid, and the ability to communicate verbally and in writing in Swedish or English. Exclusion criteria included dominating pain from other body sites than the affected knee joint, previous joint replacement or intra-articular lower limb fracture, rheumatoid arthritis, and/or neurological disease. Eleven age and gender-matched healthy controls without any known musculoskeletal disease or neurological disorders were recruited through advertisement (Table 1).

**Table 1:** Baseline characteristics, performance-based function and patient-reported outcomes in individuals with knee osteoarthritis and an age- and sex-matched control group.

Baseline characteristics	KOA group (*n* ​= ​13)	Control group (*n* ​= ​11)	Group differences
	Mean (SD)		p-value
Women, n (%) (Fishers exact)	8 (62)	8 (73)	0.679
Age	59.3 (9.4)	57.3 (10.1)	0.611
Body Mass index, kg/m^2^	24.7 (2.7)	24.3 (3.2)	0.714
Height, cm	177 (13)	170 (9.9)	0.127
Weight, kg	78.0 (15.9)	69.7 (10.3)	0.152
Previous knee arthroscopy, n (%)	8 (62)		
**Radiological classification (KL, 1**–**4), n (%)**			
1	2 (15)	–	–
2	0 (0)	–	–
3a	3 (23)	–	–
3b	4 (31)	–	–
4a	4 (31)	–	–
4b	0 (0)	–	–
**Performance-based function**			
10MWT, seconds	6.0 (0.9)	5.5 (0.6)	0.203
30STS, n of repetitions	14.5 (3.7)	20.1 (6.2)	**0.012**
SLMS, n of repetitions	21 (12.3)	32.1 (10.2)	**0.026**
JFD, cm	133 (28)	147 (49)	0.410
**Knee kinematics during performance-based tests**			
Mean ROM 30STS, degrees	78 (9)	79 (6)	0.665
ROM variation 30STS, degrees	5.4 (4.6)	7.0 (5.0)	0.437
Mean ROM SLMS, degrees	45 (8)	45 (9)	0.890
ROM variation SLMS, degrees	5.5 (4.3)	6.1 (4.6)	0.766

**Patient-reported outcome measures**	**Median (min-max)**		

**Current knee pain, visual analog scale** (0–100)	12 (1–24)	0 (0–9)	**0.012**

**KOOS** (0–100; worst-best)	**Median (min-max)**		

Pain	72 (42–100)	–	–
Symptoms	71 (43–96)	–	–
Function in ADL	88 (63–100)	–	–
Function in sport and Recreation	45 (5–95)	–	–
Knee-related quality of Life	56 (31–81)	–	–
**Forgotten joint Score-12** (0–100; worst-best)	40 (10–94)	–	–

**Pain-o-Meter**	**Median (min-max)**		

Sensory words, intensity value (0–36)	6 (0–14)	–	–
Affective words, intensity value (0–42)	6 (0–19)	–	–
**Use of analgesics, n (%)**			
Never	6 (46)	–	–
Sometimes	7 (54)	–	–
Daily	0	–	–

10MWT, 10 ​m fast-pace walk test; 30STS, 30 ​s Sit-to-Stand test; KOA; Knee osteoarthritis; SLMS, Single Leg Mini Squat test; JFD, Jump for distance; ROM, Range of motion; KOOS, Knee Injury and Osteoarthritis Outcome Score; ADL, Activities of daily living.

### 2.2 Procedures

**Fig. 1:**
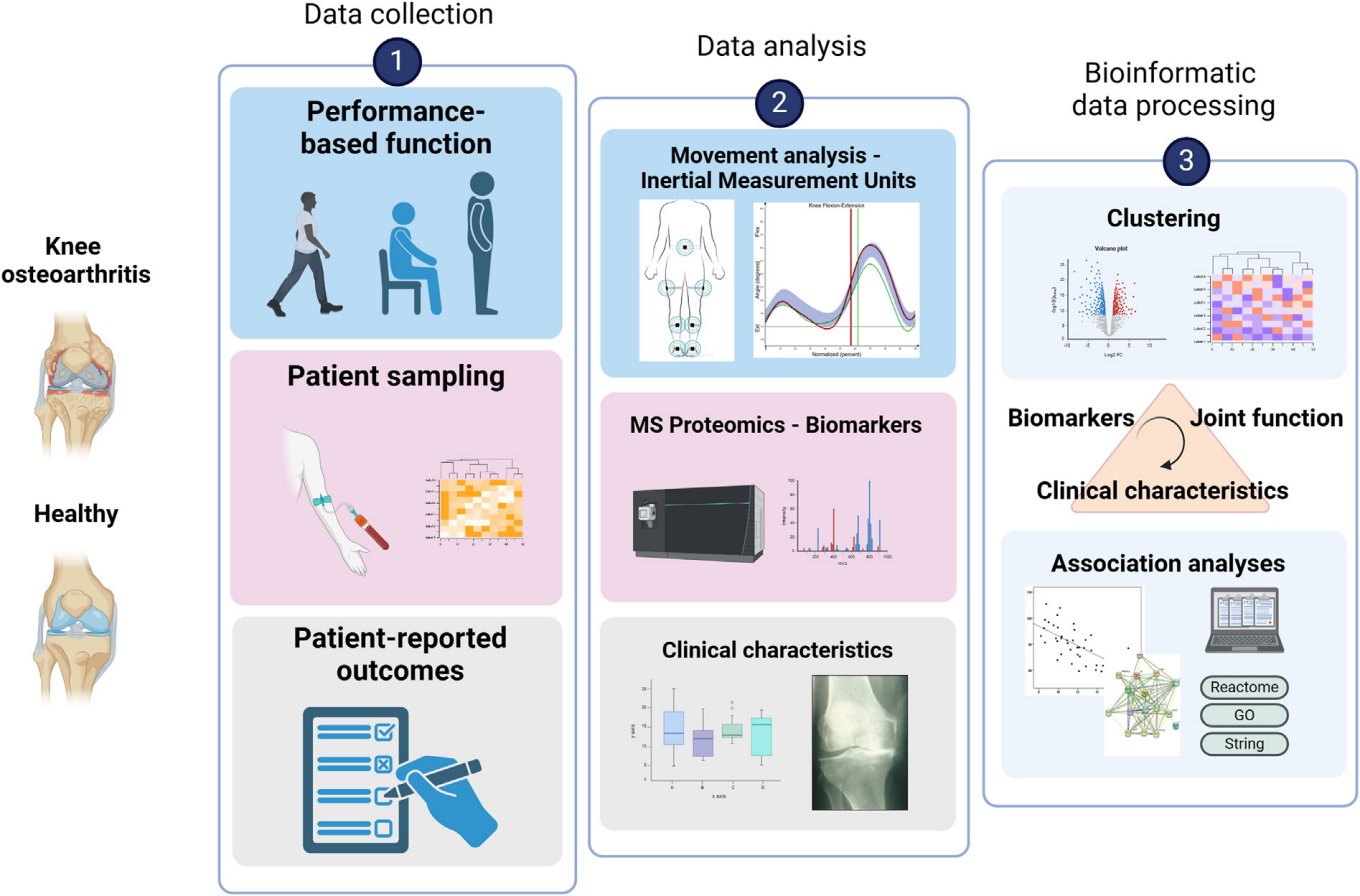
Flow chart of the BIOFUNC study. Created with BioRender.com.

All data, except the radiological examinations, were collected during one session at the Motion Analysis Lab, Karolinska University Hospital, Stockholm, Sweden. Each test session included a structured interview, a set of performance-based functional tests monitored using a sensor-based motion analysis system, blood sampling, and completion of PROM. Non-fasting blood samples were collected in EDTA tubes within 10 ​min after completion of the tests. Samples were centrifuged and the plasma frozen down in aliquots within 2 ​h from sampling and stored −80 until analyzed. The overall study design is illustrated in Fig. 1.

### 2.3 Performance-based functional tests

All participants performed the 10-m fast-paced walk test (10MWT) and 30-s Sit-to-Stand test (30STS), both of which are recommended by the Osteoarthritis Research Society International (4). After that, participants performed the Single Leg Mini Squat test (SLMS) [18,19]. Lastly, a maximal jump for distance (JFD) [20]. The 10MWT was performed twice, and the fastest trial was used for further analysis. The 30STS started with the participant standing, arms folded across their chest. The participant was instructed to sit down and stand up as many times as possible for 30 ​s (chair height 44 ​cm). The SLMS started with the participant standing on one leg on a well-defined area shaped like the letter “T”; the long axes of the foot on the stem and toes placed on the arm of the “T” [19]. A frame in front of the participant provided fingertip support for balance. The participant was instructed to flex the knee until the toes were no longer visible and then fully extend the knee [19]. Three to five practice squats preceded testing. The maximum number SLMS in 30 ​s was recorded for each leg. The maximal JFD is a reliable and valid measure of lower limb explosive power [21,22]. The participant was instructed to jump forward as far as possible, ensuring simultaneous takeoff and landing with both feet. Swinging the arms was permitted. The distance between the starting line and the toes after landing (nearest to the start line) was recorded and measured in cm. Two trials were performed, and the best trial was used for further analysis.

### 2.4 Knee joint kinematics

Knee joint kinematics were recorded during all performance-based functional tests using inertial measurement units (Opal, APDM, Portland, OR, USA) [23]. Knee kinematics from the 30STS and SLMS are included in the present study. Seven tri-axial wireless inertial unit sensors were placed using velcro-straps and double-coated adhesive tape (lumbar, things, lower legs, and feet). Knee kinematics of the sagittal plane (flexion/extension) of the entire 30-s test period of 30STS and the affected leg during the SLMS were extracted using the software Moveo Explorer by APDM (Portland, OR, USA). For the control group, the leg with the most repetitions was used for analysis. Mean knee ROM (average min-max), and within-subject variation of knee ROM, during all complete repetitions of the 30STS and SLMS were calculated and used for further analyses. MATLAB (R2022a) (The MathWorks, Inc., Natick, Massachusetts, USA) was used to automatically segment each repetition, extract the ROM, and calculate the variance.

### 2.5 Patient-reported outcome measures

All individuals included in the study rated current knee pain at the assessment time using a visual analog scale (VAS, 0–100 ​mm). In addition, all individuals with KOA completed the Knee Injury and Osteoarthritis Outcome Score (KOOS) [24], the Forgotten Joint Score-12 (FJS) [25], and the Pain-o-Meter (POM) [26]. The KOOS is a reliable measure of baseline function and change over time in individuals with KOA [27]. The KOOS is divided into five subscales: Symptoms, Pain, Function in ADL, Function in Sport and Recreation, and Knee-related Quality of Life, all of which have demonstrated adequate test-retest reliability [28]. Each subscale generates a final score ranging from 0 to 100 (0 ​= ​“worst” and 100 ​= ​“best”) [24]. The FJS is a valid and reliable questionnaire developed to assess outcomes after knee arthroplasty [25]. The final score ranges from 0 to 100 (0 ​= ​“worst” and 100 ​= ​“best”). The questionnaire has demonstrated promising results in differentiating well-functioning individuals [25]. The POM is a validated instrument used to describe pain in patients with different chronic diseases [26,29,30]. The instrument consists of two components; the present study used one of the components, namely POM-Words, which consists of 12 sensory- and 11 affective words to describe pain qualitatively. An intensity score is assigned to each word, unknown to the person rating their pain, ranging from 1 to 5 (1 is considered a lighter pain than 5). The values are summed to one intensity score: for sensory words and one for affective words. Individuals with OA were instructed to choose as many words from the sensory- and affective groups as necessary to describe their knee pain.

### 2.6 Radiological examination

Weight bearing anterior-posterior radiographs of the tibiofemoral joint (with extended knees), no older than six months from baseline testing, were classified according to the modified Kellgren and Lawrence’s classification (KL) ranging from grade I-IV [31]. Radiographs, defined as KL scores of 3–4, were further sub-classified by incorporating scores of joint space narrowing (JSN) and bone attrition [32]. Thus, a KL grade 3 radiograph with mild JSN was graded 3a, and radiographs with more severe JSN 3b. A KL grade 4 radiograph demonstrating complete loss of joint space was divided into 4a if no bone attrition and 4b if subchondral bone attrition existed. Three assessors classified all radiographs.

### 2.7 Protein extraction and digestion of plasma samples

In blood plasma samples SureQuant500 quantitative MS was used to identify DEPs in patients compared to controls (4). An aliquot of 10 ​μL patient blood plasma sample, supplemented with 10 ​μL of 8 ​M urea in 50 ​mM ammonium bicarbonate (AmBic), was used for protein digestion, followed by the addition of 10 ​μL of 0.2 ​% ProteaseMAX (Promega) in 20 ​% acetonitrile (AcN) and 50 ​mM AmBic. Additional 220 ​μL of 50 AmBic was added before determining the protein concentrations by BCA assay (Pierce). An aliquot of 25 ​μg proteins was reduced with 10 ​μL of 100 ​mM dithiothreitol in 50 ​mM AmBic, incubated at 37 ​°C and alkylated with 20 ​μL of 100 ​mM iodoacetamide in 50 ​mM AmBic before incubation at the room temperature in dark. Proteolytic digestion was performed by adding 2.5 ​μL of 0.2 ​μg/μL sequencing grade trypsin (Promega) incubated at 37 ​°C overnight. The reaction was stopped with 5 ​μL of concentrated formic acid, and samples were cleaned on a C18 Hypersep plate with a bed volume of 40 ​μL (Thermo Scientific) and dried in a vacuum concentrator (Eppendorf).

### 2.8 Reversed-phase liquid chromatography-tandem mass spectrometry (RPLC-MS/MS) analysis

PQ500 reference mixture (Biognosys, Switzerland) with 804 stable isotope labeled (SIL) peptides was prepared according to the manufacturer’s instructions, adding 20 ​μL of dissolution buffer and diluted with 100 ​μL of solvent A (2 ​% AcN, 0.1 ​% formic acid). Blood plasma digests were dissolved in solvent A to obtain a final concentration of 0.88 ​μg/μL. The SIL standard peptides were spiked into the patient samples in 4 ​μL volumes. The RPLC was performed on a 15 ​cm long C18 EASY-spray and C18 trap columns connected to an Ultimate 3000 UPLC system (Thermo Fisher Scientific). *Mass spectra* were acquired on an Orbitrap Fusion Lumos tribrid mass spectrometer (Thermo Fisher Scientific) targeting 10^6^ ions in a maximum of 50 ​ms for the survey method. An inclusion mass list generated in Skyline Daily v20.1.1.196 (MacCoss Laboratory, University of Washington, Seattle, WA) targeted selected precursors and fragmentized them by higher-energy collisional dissociation. The tandem mass spectra were acquired with a resolution of *R* ​= ​7,500, targeting 5 ​× ​10^5^ ions in a maximum 10 ​ms injection time. After adjusting the intensity thresholds of each precursor in Skyline, the SureQuant PQ500 method workflow was used to acquire quantitative data. The tandem mass spectra were acquired with an isolation offset dictated by the mass difference between the SIL and the endogenous peptides. *The raw files* were imported to Skyline Daily and targeted proteins were quantified relatively by comparing SIL and endogenous peptides. Both the survey and SureQuant methods included 804 peptides of 581 human proteins in total. The mean intensity ratios of endogenous and SIL peptides were used for further analyses when multiple peptides were available for a protein.

### 2.9 Data reduction and statistical analysis

All data analyzed using SPSS statistics (IBM, Version 26 Armonk, NY, USA). The statistical significance level was set at *ɑ* ​= ​0.05. The normality of data was assessed with Q-Q plots and Shapiro-Wilk’s test. Descriptive data were reported as mean, standard deviation, frequency, median, and range. *Principal Component Analysis (PCA) and heatmap clustering* were analyzed based on all DEPs (http://www.heatmapper.ca/expression/). Values were scaled by row, and distance was measured by Spearman rank correlation. Rows and columns were clustered by complete linkage clustering. *Linear correlations* were used to explore associations between DEPs (up- and downregulated) and clinical outcome measures (performance-based tests, knee kinematics, and PROM). The associations were determined by using Spearman’s Rho. To analyze dysregulated pathways *enrichment analysis* of all DEPs was performed against Reactome, version 87, without the inclusion of interactors (http://reactome.org/). Pathways with p-values <0.05 were considered significantly enriched.

## 3 Results

### 3.1 Reduced function and increased knee pain in individuals with KOA

Thirteen individuals with KOA and eleven healthy controls were included in the study. Their clinical characteristics are described in Table 1. Individuals with KOA performed fewer repetitions during the 30STS and SLMS than controls. However, no differences were found in time to complete the 10MWT or JFD (Table 1). When comparing groups, kinematic analyses did not detect differences in mean knee ROM or ROM variation during the 30STS and SLSM (Table 1). Individuals with KOA reported a higher level of current knee pain using VAS (Table 1).

### 3.2 Distinct proteomic profile in KOA

**Fig. 2:**
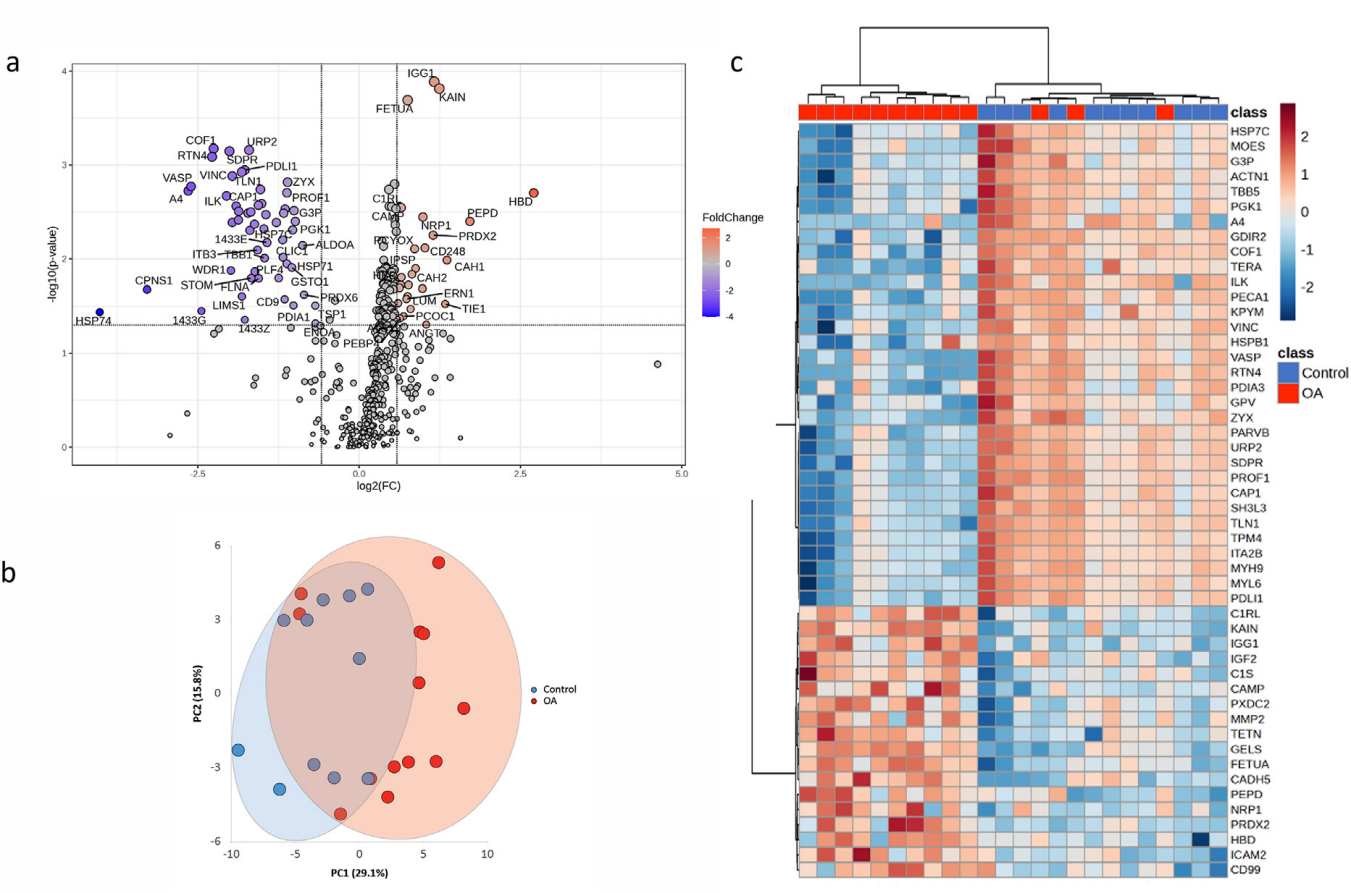
Proteomic Profiling of knee osteoarthritis (KOA). Analysis of the proteomic profile identified 82 DEPs, of which 28 were significantly upregulated and 54 were significantly downregulated in KOA compared to controls, displayed in the volcano plot in (a). Principal component analysis (b) distinguished two major clusters with overlap between groups, similar to hierarchical cluster analysis (c) where KOA and healthy separated based on their protein expression patterns.

MS analysis identified a proteomic landscape of 321 (of 581) proteins across all samples and 458 proteins when using a detection cut-off that the protein should be detected in at least 50 ​% of all samples (KOA and controls). Data analysis of the proteomic profile, using the 50 ​% cut-off identified 82 DEPs, of which 54 were significantly downregulated and 28 significantly upregulated in KOA compared to healthy individuals. (Fig. 2A). Hierarchical cluster analysis distinguished two major clusters with some overlap (Fig. 2B), similar to PCA, which displayed distinct features between KOA and controls (Fig. 2C).

### 3.3 Associations between DEPs, performance-based joint function, and PROM

**Fig. 3:**
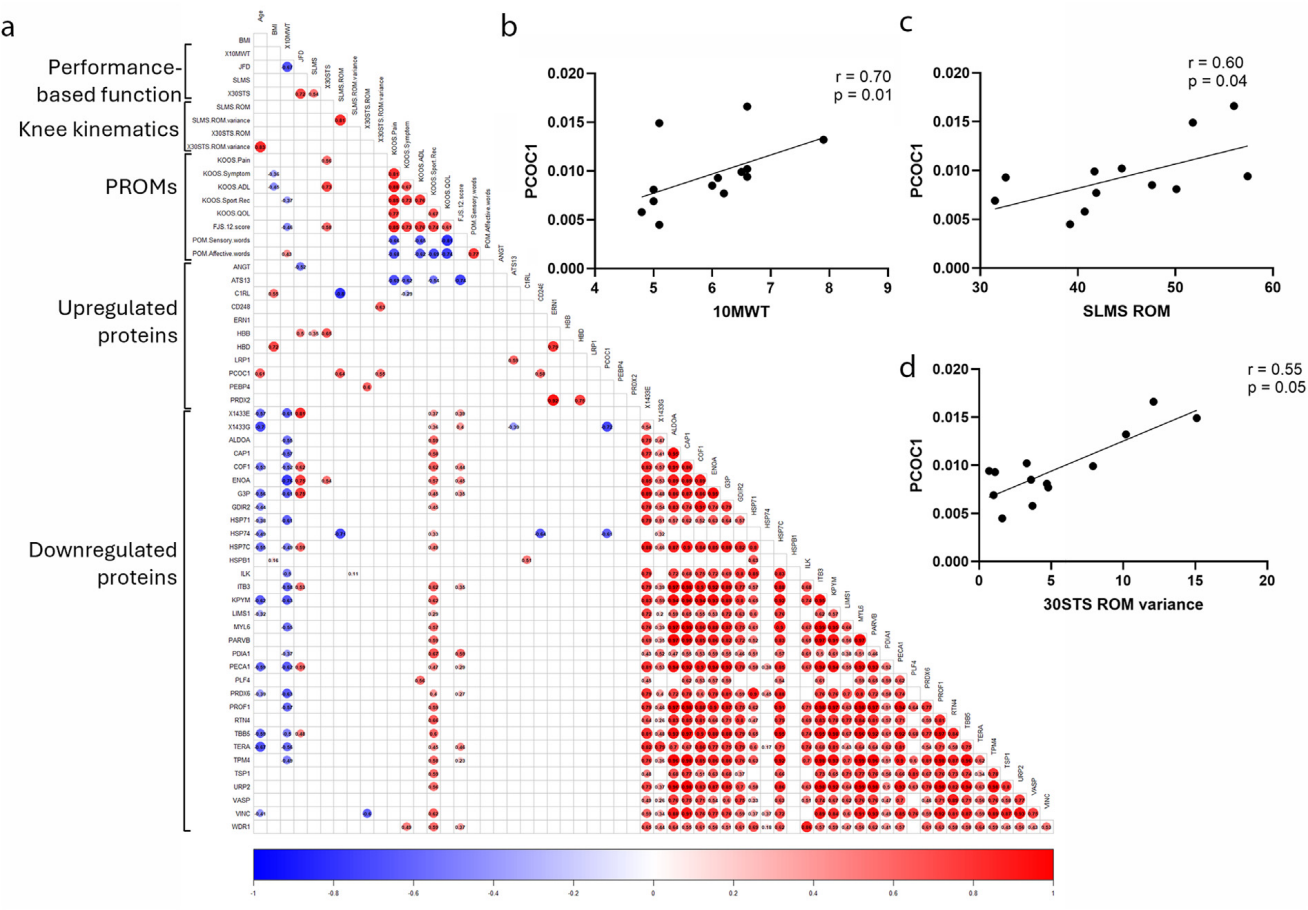
Color matrix of the association analyses between a) Performance-based functional tests, knee kinematics, patient-reported outcome measures (PROM), upregulated- and downregulated DEPs. Linear correlations between PCOC1 and b) 10 ​m fast-paced walk test, c) Knee joint ROM during the SLMS, and d) Knee joint range ROM variance during the 30-s Sit-to-Stand test (30STS).

Two upregulated proteins from hemostasis pathway (ATS13, HBD) associated with patient-reported pain and symptoms (KOOS subscales and FJS). In addition, PRDX2 was associated with KOOS Pain, while ERN1 and LRP1 were associated with FJS. Twenty of the downregulated proteins were associated with KOOS Sport and Recreation (Fig. 3A and Supplementary Table S2).

### 3.4 Enriched pathways display platelets, extracellular matrix and neutrophil dysregulation

**Fig. 4:**
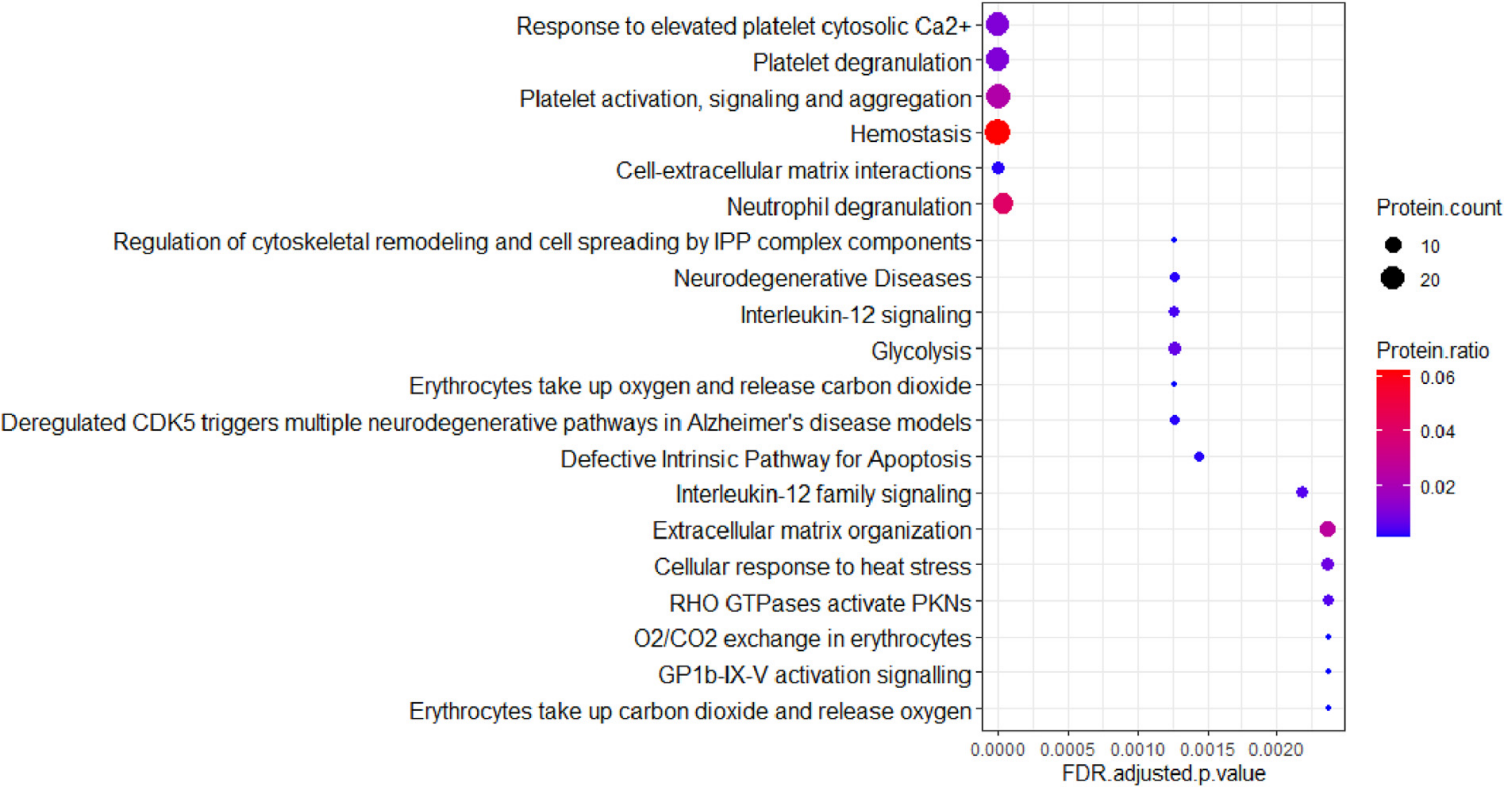
Top 20 pathways via Reactome enrichment analysis.

Reactome enrichment analysis of all DEPs displayed 102 significantly enriched pathways (Supplementary Table S1). Platelet activation and degranulation, extracellular matrix (ECM)-interactions, and neutrophil degranulation were among the top 20 enriched pathways (Fig. 4).

A total of 23 DEPs were involved in platelet activation and platelet degranulation pathways. Four of 23 DEPs (COF1, PECA1, TSP1 and VINC) were associated with performance-based function or kinematics, and ten (ACTN1, ALDOA, CAP1, COF1, ITB3, PLF4, PROF1, TSP1, VINC and WDR1) with PROM, in particular KOOS Sport and Recreation (Fig. 3A and Supplementary Table S2).

Fifteen DEPs were involved in ECM organization and cell-ECM organization, supporting the role of ECM dysregulation in KOA. Five DEPs (ILK, LIMS1, PCOC1, PECA1 and TSP1) from ECM pathways were associated with performance-based function or kinematics, and six (ACTN1, ILK, ITB3, PARVB, PDIA1 and TSP1) with PROM, in particular KOOS Sport and Recreation (Fig. 3A–D and Supplementary Table S2).

Sixteen DEPs were involved in neutrophil degranulation, and together with the enriched pathways of IL12 and IL12 signaling, this indicates presence of inflammation in OA. Eight DEPs (HBB, HSPB1, HSP71, HSP7C, KPYM, PECA1, PRDX6, TERA and VINC) from neutrophil pathway were associated with performance-based function or kinematics, and four (ALDOA, CAP1, TBB5 and VINC) with PROM, in particular KOOS Sport and Recreation (Fig. 3A and Supplementary Table S2).

## 4 Discussion

This cross-sectional study examined the plasma proteomic landscape of KOA and identified 82 DEPs, of which 44 were related to performance-based function, knee kinematics, and/or PROM. Our findings indicate that clinical determinants of KOA, including both knee joint function and pain, are associated with specific serological biomarkers of platelet degranulation, ECM organization, and neutrophil degranulation. Further exploration of protein characteristics and the associations with joint function and pain may provide novel biomarkers and specific functional proteins for targeted treatment of KOA.

As expected, individuals with KOA reported higher levels of current knee pain and demonstrated reduced performance-based function, as measured by the number of STS and SLMS repetitions performed. Pain and symptoms were reported at lower levels compared to Swedish registry data for KOA (KOOS) [33]. These findings suggest that reductions in the maximal performance-based function (i.e., capacity) may manifest earlier than the onset of patient-reported pain and symptoms. In the present study we used various performance-based tests to capture multiple aspects of joint function, evaluating explosive capacity (jump), the ability to quickly change between concentric and eccentric muscle activity, and functional mobility. Reduced walking speed is a strong predictor of mortality [34] and risk of knee joint replacement [35]. The 10MWT and PROM both aligned with DEP profiles. Knee joint kinematics, which capture movement quality, could detect early signs of joint disease. The 10MWT and PROM both align with DEPs profiles. This study suggests that knee joint kinematics could be used to capture movement quality and detect early signs of joint disease.

Contrary to our expectations, all ROM variables were negatively correlated with downregulated proteins. ROM variation during the SLMS, a one-leg performance-based test that can be compared to stair negotiation, was negatively correlated to three downregulated DEPs (ANGT, C1RL, LRP1). A small variation in ROM may indicate poorer joint function, suggesting limited movement patterns of KOA joints [36]. Further evaluation in a larger sample is needed to fully understand if a smaller ROM variation is a clinical characteristic in individuals with KOA. In addition, investigations of compensatory movement patterns in adjacent and contralateral joints are necessary to understand these features.

Five upregulated proteins (ATS13, ERN1, HBD, LRP1, PRDX2) were associated with pain-related PROM. Individuals with KOA who reported increased pain and symptoms (measured by KOOS Pain, KOOS Symptoms, and FJS) exhibited lower levels of ATS13, a metalloprotease known to act exclusively on von Willebrand factor. This finding aligns with a mouse study in which treatment with rADAMTS13 demonstrated anti-inflammatory effects, and ADAMTS-13 knock-out mice were protected from arthritis [37]. However, this contradicts our observation that individuals with KOA exhibited higher levels of ATS13 compared to healthy controls. These findings may suggest a novel role of ATS13 in KOA-related pain and joint function, warranting further investigation.

Reactome enrichment analysis of DEPs highlighted pathways related to platelets, ECM, and neutrophils among the top 20 pathways. Twenty-three DEPs were involved in platelet activation and platelet degranulation pathways, indicating a role for platelets in KOA pathogenesis. In the KOA group, 54 ​% (7 of 13) reported occasional use of pain medication (“sometimes”; Table 1) with two individuals using non-steroidal anti-inflammatory drugs. Non-steroidal anti-inflammatory drugs are known to produce a mild, systemic hemostatic defect by inhibiting normal platelet function [38]. Intra-articular injections of platelet-rich plasma are in clinical use, although controversial, with limited evidence and unknown modes of action [39,40].

Platelet endothelial cell adhesion molecule (PECAM-1) was downregulated in KOA and associated with performance-based function, where lower levels of PECAM-1 correlated to slower walking speed and shorter jump distance. PECAM-1 is important for leukocyte *trans*-endothelial migration during inflammation and is associated with angiogenesis, which is implicated in OA progression [41,42]. No pain associations with PECAM-1 were detected in this dataset, although development and formation of new blood vessels are associated with nerve ingrowth, which could influence pain. At least ten DEPs (ACT, CAP1, COF1, FLNA, PARVB, PROF1, TLN1, VINC, WDR1, ZYX) are involved in actin cytoskeleton changes, with the ZYX-ACT-VINC axis playing a key role in collagen synthesis and cytoskeleton-ECM remodeling in cartilage [43]. This axis was downregulated in KOA, with lower levels correlated to worse performance-based function. COF-1 and VINC, both associated with chondrocyte metabolism and ECM organization, were also linked to worse performance-based function and PROM (KOOS Sport Recreation) [[43], [44], [45], [46], [47]]. The role of actin regulatory proteins in KOA requires further exploration to elucidate the mechanisms of inflammation and cartilage structure.

Structural changes in bone and cartilage, confirmed by enriched ECM-related pathways in KOA patients, were observed. Proteins such as PCOC1, PECAM-1, and TSP1 correlated with performance-based function, knee kinematics, and pain parameters. PCOC1, involved in collagen turnover, may affect joint tissue biomechanical properties [48,49]. Our data suggests that the increased PCOC1 levels observed in individuals with KOA may indicate an imbalance in collagen degradation and production. Collagen turnover and the role of PCOC1 in KOA would be interesting to follow up in future studies.

TSP1, an adhesive glycoprotein mediating cell interactions and protecting cartilage, was downregulated in KOA and associated with worse KOOS Sport and Recreation scores. The association of TSP1 with collagens as an integral part of ECM structural proteins is well established [50]. TSP1 is expressed by chondrocytes and is believed to protect the cartilage by inhibiting proteases such as elastase and cathepsin B [[51], [52], [53]]. Further, it acts as an angiogenesis inhibitor and has been demonstrated to suppress disease progression in a rat model of OA (ACLT) [50]. The ability of TSP1 to inhibit a broad spectrum of proteases may enable it to protect and stabilize ECM during tissue remodeling. Decreased TSP1 levels may negatively impact cartilage tissue. Lower TSP1 levels were paradoxically linked to larger ROM, indicating the need for further investigation into TSP1’s effects on joint function.

To our knowledge, this is the first study to explore the associations between the plasma proteomic landscape of KOA and clinical manifestations using both objective performance-based tests and PROM. The well-characterized cohort, age and sex-matched healthy controls, comprehensive clinical data, and use of targeted MS with internal standards for protein quantification provide robust data. Study limitations include a small sample size and sex bias towards women. SureQuant PQ500 MS utilizes internal standards which limits the number of proteins that can be detected, and the following enrichment analysis for activated pathways is also restricted to the pre-chosen panel.

In conclusion, individuals with KOA differed from controls across all three assessment modalities, where individuals with KOA presented with worse joint function, higher levels of pain, and expressed different proteins in plasma. We observed multiple associations between DEPs, joint function, and PROM. The described study protocol, combining biomarker evaluation, joint function, and PROM, shows promise for future multivariate analyses to subgroup individuals with KOA.

## Ethics approval and consent to participate

This study was approved by the Swedish Ethical Review Authority, DNR: 2019-02009.

## Consent for publication

N/A.

## Availability of data and material

The datasets used and analyzed for this study are not publicly available due to ethical concerns but are available from the corresponding author on reasonable request.

## Authors' contributions

JEN, CA: Conception and study design; JEN, CA, AV: data collection; JEN, CA, AA, MH, MBS, AV: data analysis; JEN, CA, AA: interpretation of data, JEN, CA: drafting the manuscript; All authors: participated in critical revision of the manuscript; JEN, CA, HEH, EWB: Funding; All authors read and approved the final submitted manuscript.

## Funding

This study was supported by grants provided by Strategiska forskningsområdet vårdvetenskap (SFO-V) Karolinska Instiutet, Alex och Eva Wallströms stiftelse, Magnus Bergvalls stiftelse, af Ugglas stiftelse, 10.13039/100009389Stiftelsen Promobilia, Stiftelsen skobranschens utvecklingsfond, Loo och Hans Ostermans stiftelse, and Angeby stiftelse. Josefine E Naili was supported by Region Stockholm (clinical research appointment).

The funders had no role in study design, data collection and analysis, interpretation of data, decision to publish, or preparation of the manuscript.

## Declaration of competing interest

Each author certifies that there is no commercial associations that might pose a conflict of interest in connection with the submitted article.
