# Supplementary material for: Proteomic analysis reveals biomarkers associated with performance-based joint function and patient-reported outcomes in knee osteoarthritis
Source: Osteoarthr Cartil Open. 2024 Nov 16;7(1):100543. doi: 10.1016/j.ocarto.2024.100543 (PMC11616498; doi:10.1016/j.ocarto.2024.100543)
Supplement: Multimedia component 1 [file mmc1.pdf]

Pathway name	#Entities found	#Entities total	Entities ratio	Entities pValue	Entities FDR	#Reactions found	#Reactions total	Reactions ratio
Platelet degranulation	21	128	0.011	0,0000	0.00000000000002	5	11	0.001
Response to elevated platelet cytosolic Ca2+	21	133	0.011	0,0000	0.00000000000002	5	14	0.001
Platelet activation. signaling and aggregation	23	265	0.023	0,0000	0.00000000000002	34	119	0.008
Hemostasis	28	727	0.062	0,0000	0.00000000002365	59	342	0.024
Cell-extracellular matrix interactions	6	18	0.002	0,0000	0.00000055209136	7	10	0.001
Neutrophil degranulation	16	478	0.041	0.0000004	0.00003387149408	9	10	0.001
Neurodegenerative Diseases	4	22	0.002	0.0000243	0.00125723345355	7	22	0.002
Deregulated CDK5 triggers multiple neurodegenerative pathways in Alzheimer's disease models	4	22	0.002	0.0000243	0.00125723345355	7	22	0.002
Interleukin-12 signaling	5	46	0.004	0.0000263	0.00125723345355	6	56	0.004
Glycolysis	6	80	0.007	0.0000317	0.00125723345355	5	24	0.002
Regulation of cytoskeletal remodeling and cell spreading by IPP complex components	3	8	0.001	0.0000322	0.00125723345355	3	5	0,0000
Erythrocytes take up oxygen and release carbon dioxide	3	8	0.001	0.0000322	0.00125723345355	2	6	0,0000
Defective Intrinsic Pathway for Apoptosis	4	25	0.002	0.0000399	0.00143705222126	7	24	0.002
Interleukin-12 family signaling	5	56	0.005	0.0000663	0.00218678326133	7	114	0.008
Extracellular matrix organization	10	300	0.026	0.0000796	0.00236189613864	38	319	0.022
Cellular response to heat stress	6	95	0.008	0.0000814	0.00236189613864	14	29	0.002
RHO GTPases activate PKNs	5	62	0.005	0.0001065	0.00236679166009	2	20	0.001
GP1b-IX-V activation signalling	3	12	0.001	0.0001065	0.00236679166009	7	9	0.001
Erythrocytes take up carbon dioxide and release oxygen	3	12	0.001	0.0001065	0.00236679166009	4	8	0.001
O2/CO2 exchange in erythrocytes	3	12	0.001	0.0001065	0.00236679166009	6	14	0.001
Glucose metabolism	6	100	0.009	0.0001076	0.00236679166009	9	49	0.003
Semaphorin interactions	5	64	0.005	0.0001233	0.00259029032134	17	41	0.003
Chk1/Chk2(Cds1) mediated inactivation of Cyclin B:Cdk1 complex	3	13	0.001	0.0001347	0.00269346699292	1	5	0,0000
Gluconeogenesis	4	35	0.003	0.0001449	0.00275404550841	4	25	0.002
Attenuation phase	3	14	0.001	0.0001673	0.00275886548879	3	5	0,0000
Signaling by high-kinase activity BRAF mutants	4	37	0.003	0.000179	0.00275886548879	4	6	0,0000
Gene and protein expression by JAK-STAT signaling after Interleukin-12 stimulation	4	37	0.003	0.000179	0.00275886548879	4	36	0.003
p130Cas linkage to MAPK signaling for integrins	3	15	0.001	0.0002047	0.00275886548879	3	3	0,0000
GRB2:SOS provides linkage to MAPK signaling for Integrins	3	15	0.001	0.0002047	0.00275886548879	2	2	0,0000
Activation of BAD and translocation to mitochondria	3	15	0.001	0.0002047	0.00275886548879	2	5	0,0000
SARS-CoV-2 targets host intracellular signalling and regulatory pathways	3	15	0.001	0.0002047	0.00275886548879	1	5	0,0000
Translocation of SLC2A4 (GLUT4) to the plasma membrane	5	72	0.006	0.0002122	0.00275886548879	4	15	0.001
Detoxification of Reactive Oxygen Species	4	39	0.003	0.0002185	0.00284036028661	4	32	0.002

Pathway name	#Entities found	#Entities total	Entities ratio	Entities pValue	Entities FDR	#Reactions found	#Reactions total	Reactions ratio
Axon guidance	13	558	0.048	0.0002395	0.00287366838904	33	297	0.021
Platelet Aggregation (Plug Formation)	4	40	0.003	0.0002404	0.00287366838904	22	27	0.002
Manipulation of host energy metabolism	2	3	0,0	0.0002413	0.00287366838904	2	2	0,0000
Regulation of HSF1-mediated heat shock response	5	75	0.006	0.0002558	0.00287366838904	8	14	0.001
Cell junction organization	6	118	0.01	0.0002612	0.00287366838904	7	68	0.005
MAP2K and MAPK activation	4	41	0.003	0.0002639	0.00290278963603	8	12	0.001
Signaling by RAF1 mutants	4	42	0.004	0.000289	0.00298404641209	4	7	0,0000
L1CAM interactions	6	121	0.01	0.0002984	0.00298404641209	10	54	0.004
Smooth Muscle Contraction	4	44	0.004	0.0003442	0.00333325203699	5	16	0.001
RHO GTPase Effectors	9	294	0.025	0.0003444	0.00333325203699	19	113	0.008
SARS-CoV-1 targets host intracellular signalling and regulatory pathways	3	18	0.002	0.0003481	0.00333325203699	1	7	0,0000
Nervous system development	13	584	0.05	0.0003704	0.00333325203699	33	323	0.023
RHO GTPases Activate ROCKs	3	19	0.002	0.0004072	0.00361142190835	3	7	0,0000
Signaling downstream of RAS mutants	4	47	0.004	0.0004406	0.00361142190835	4	7	0,0000
Signaling by moderate kinase activity BRAF mutants	4	47	0.004	0.0004406	0.00361142190835	4	7	0,0000
Paradoxical activation of RAF signaling by kinase inactive BRAF	4	47	0.004	0.0004406	0.00361142190835	4	7	0,0000
Signaling by RAS mutants	4	47	0.004	0.0004406	0.00361142190835	4	9	0.001
Integrin cell surface interactions	5	85	0.007	0.0004514	0.00361142190835	19	55	0.004
Signaling by Rho GTPases	14	677	0.058	0.000453	0.0036237289622	36	203	0.014
RHO GTPases activate PAKs	3	21	0.002	0.0005439	0.00435138954667	5	15	0.001
Signal transduction by L1	3	21	0.002	0.0005439	0.00435138954667	2	11	0.001
Signaling by Rho GTPases. Miro GTPases and RHOBTB3	14	693	0.059	0.0005708	0.00456607135981	36	212	0.015
Chaperone Mediated Autophagy	3	22	0.002	0.0006221	0.00497645216337	12	19	0.001
HSF1-dependent transactivation	3	24	0.002	0.000799	0.00559318399745	4	8	0.001
HSP90 chaperone cycle for steroid hormone receptors (SHR) in the presence of ligand	4	57	0.005	0.0009011	0.00630776790889	9	22	0.002
Innate Immune System	19	1197	0.102	0.001089	0.00762318732722	20	690	0.048
Cell-Cell communication	6	156	0.013	0.0011159	0.00781115491501	8	91	0.006
Syndecan interactions	3	27	0.002	0.0011196	0.00783736880879	4	15	0.001
Integrin signaling	3	28	0.002	0.0012421	0.00869438849899	21	24	0.002
Activation of BH3-only proteins	3	30	0.003	0.0015115	0.01005062025689	2	19	0.001
Signaling by BRAF and RAF1 fusions	4	66	0.006	0.0015401	0.01005062025689	4	5	0,0000
Defects of platelet adhesion to exposed collagen	2	8	0.001	0.0016751	0.01005062025689	4	5	0,0000
Cellular responses to stress	14	784	0.067	0.0018596	0.01115733396256	43	492	0.034
CHL1 interactions	2	9	0.001	0.0021099	0.01265928049326	2	5	0,0000
Late endosomal microautophagy	3	34	0.003	0.0021539	0.01292366362689	3	3	0,0000
Cellular responses to stimuli	14	798	0.068	0.0021902	0.01314098438406	43	523	0.036
Immune System	28	2219	0.189	0.002204	0.01322392367512	45	1668	0.116
ECM proteoglycans	4	76	0.006	0.0025607	0.01536430676382	5	23	0.002
RHOH GTPase cycle	3	37	0.003	0.002732	0.01639189386573	2	3	0,0000
Diseases of programmed cell death	4	79	0.007	0.00294	0.01763974745242	7	37	0.003
Formation of Fibrin Clot (Clotting Cascade)	3	39	0.003	0.0031656	0.01827280202223	10	61	0.004
PKR-mediated signaling	4	81	0.007	0.0032131	0.01827280202223	3	40	0.003
Oncogenic MAPK signaling	4	84	0.007	0.0036546	0.01827280202223	24	46	0.003

Pathway name	#Entities found	#Entities total	Entities ratio	Entities pValue	Entities FDR	#Reactions found	#Reactions total	Reactions ratio
PECAM1 interactions	2	12	0.001	0.0036972	0.0184861078548	7	7	0,0000
Regulation of localization of FOXO transcription factors	2	12	0.001	0.0036972	0.0184861078548	3	5	0,0000
HSF1 activation	2	12	0.001	0.0036972	0.0184861078548	2	7	0,0000
Reversible hydration of carbon dioxide	2	12	0.001	0.0036972	0.0184861078548	2	8	0.001
TP53 Regulates Metabolic Genes	4	88	0.008	0.0043049	0.0215246251416	5	34	0.002
Regulation of mRNA stability by proteins that bind AU-rich elements	4	88	0.008	0.0043049	0.0215246251416	3	26	0.002
SEMA3A-Plexin repulsion signaling by inhibiting Integrin adhesion	2	14	0.001	0.0049843	0.02492135449876	7	8	0.001
Signaling by ALK fusions and activated point mutants	4	93	0.008	0.0052224	0.02611222089644	3	63	0.004
Signaling by ALK in cancer	4	93	0.008	0.0052224	0.02611222089644	3	71	0.005
Cellular response to chemical stress	6	216	0.018	0.0055364	0.02768214156323	13	166	0.012
Autophagy	5	152	0.013	0.0056151	0.02807532585006	25	108	0.008
Recruitment of NuMA to mitotic centrosomes	4	95	0.008	0.0056234	0.02811724221964	2	2	0,0000
Sema3A PAK dependent Axon repulsion	2	16	0.001	0.006448	0.02897750193528	4	6	0,0000
Platelet Adhesion to exposed collagen	2	16	0.001	0.006448	0.02897750193528	2	8	0.001
Protein methylation	2	17	0.001	0.0072444	0.02897750193528	2	9	0.001
Antiviral mechanism by IFN-stimulated genes	5	162	0.014	0.0072922	0.02916885281178	4	71	0.005
Metabolism of carbohydrates	7	304	0.026	0.0075571	0.03022844454233	19	234	0.016
Disease	26	2188	0.187	0.007607	0.03042786749316	72	1905	0.133
Intrinsic Pathway for Apoptosis	3	55	0.005	0.0081574	0.03262940354674	2	63	0.004
AUF1 (hnRNP D0) binds and destabilizes mRNA	3	56	0.005	0.0085651	0.03426032873381	2	4	0,0000
Diseases of hemostasis	2	19	0.002	0.008963	0.03585208874157	5	23	0.002
Non-integrin membrane-ECM interactions	3	59	0.005	0.0098593	0.03943729394411	4	22	0.002
RHO GTPases activate CIT	2	20	0.002	0.009884	0.03953584964697	1	6	0,0000
Sema4D induced cell migration and growth-cone collapse	2	20	0.002	0.009884	0.03953584964697	1	7	0,0000
Common Pathway of Fibrin Clot Formation	2	22	0.002	0.0118459	0.04738354962798	5	29	0.002
RUNX1 regulates genes involved in megakaryocyte differentiation and platelet function	3	64	0.005	0.012257	0.04902814137149	3	33	0.002
