# Supplementary material for: Proteomic analysis reveals biomarkers associated with performance-based joint function and patient-reported outcomes in knee osteoarthritis
Source: Osteoarthr Cartil Open. 2024 Nov 16;7(1):100543. doi: 10.1016/j.ocarto.2024.100543 (PMC11616498; doi:10.1016/j.ocarto.2024.100543)
Supplement: Multimedia component 2 [file mmc2.pdf]

UPREGULATED		Performance-based function				Knee kinematics				Patient-reported outcome measures							
		10MWT	JFD	SLMS	30STS	SLMS ROM	SLMS ROM variance	30STS ROM	30STS ROM variance	KOOS Pain	KOOS Symptom	KOOS ADL	KOOS Sport/Rec	KOOS QOL	FJS-12 score	POM Sensory-words	POM Affective-words
	ANGT						r=-0.587 p=0.045										
	ATS13									r=-0.687 p=0.009	r=-0.618 p=0.024				r=-0.737 p=0,004		
	C1RL					r=-0.797 p=0.002	r=-0.622 p=0,031										
	CD248								r=0.632 p=0.021								
	ERN1												r=-0.,561 p=0.046		r=-0.572 p=0,041		
	HBB				r=0.646 p=0.017												
	HBD									r=-0.630 p=0.021		r=-0.619 p=0.024	r=-0.555 p=0.049		r=-0.642 p=0,018		
	LRP1						r=-0.681 p=0,015								r=-0.577 p=0,039		
	PCOC1	r=0.663 p=0.014				r=0.636 p=0.026			r=0.555 p=0.049								
	PEBP4							r=0.602 p=0,029									
	PRDX2									r=-0.556 p=0.049							

DOWNREGULATED		Performance-based function				Knee kinematics				Patient-reported outcome measures							
		10MWT	JFD	SLMS	30STS	SLMS ROM	SLMS ROM variance	30STS ROM	30STS ROM variance	KOOS Pain	KOOS Symptom	KOOS ADL	KOOS Sport/Rec	KOOS QOL	FJS-12 score	POM Sensory-words	POM Affective-words
	1433E	r=-0.586 p=0.035	r=0.806 p=0.001														
	1433G	r=-0.607 p=0.028															
	ACTN1												r=0.659 p=0.014				
	ALDOA												r=,0.586 p=0.035				
	CAP1												r=0.585 p=0.036				
	COF1		r=0.618 p=0.024										r=0.624 p=0.023				
	ENOA	r=-0.726 p=0.005	r=0.753 p=0.003										r=0.571 p=0.042				
	G3P	r=-0.567 p=0.043	r=0.751 p=0.003		r=0.568 p=0.043												
	GDIR2		r=0.567 p=0.043														
	HSP71	r=-0.610 p=0.027	r=0.583 p=0.037														
	HSP74					r=-0.706 p=0.01	r=-0.832 p=0.001										
	HSP7C		r=0.588 p=0.035														
	HSPB1	r=-0.675 p=0.011	r=0,653 p=0.016														

C

DOWNREGULATED

	Performance-based function				Knee kinematics				Patient-reported outcome measures							
	10MWT	JFD	SLMS	30STS	SLMS ROM	SLMS ROM variance	30STS ROM	30STS ROM variance	KOOS Pain	KOOS Symptom	KOOS ADL	KOOS Sport/Rec	KOOS QOL	FJS-12 score	POM Sensory-words	POM Affective-words
ILK		r=0,624 p=0.023										r=0.558 p=0.048				
ITB3												r=0.617 p=0.025				
KPYM	r= -0.591 p=0.033	r=0.560 p=0.046										r=0.615 p=0.025				
LIMS1							r=-0.556 p=0.049									
MYL6												r=0.574 p=0.04				
PARVB												r=0.588 p=0.034				
PDIA1												r=0.675 p=0.011		r=0.586 p=0.035		
PDLI1												r=0.564 p=0.045				
PECA1	r=-0.572 p= 0.041	r=0.588 p=0.035														
PLF4											r=0.563 p=0.045					
PRDX6	r=-0,609 p=0.027	r=0.613 p=0.026														
PROF1												r=0.588 p=0.035				
RTN4												r=0.657 p=0.015				
TBB5												r=0.596 p=0.032				
TERA		r=0.590 p=0.034														
TPM4												r=0.582 p=0.037				
TSP1							r=-0.676 p=0.011					r=0.588 p=0.035				
URP2												r=0.561 p=0.046				
VINC							r=-0.602 p=0.029					r=0.620 p=0.024				
WDR1												r=0.588 p=0.035				
